# Selective facet stabilization of anatase TiO_2_ by Sn doping

**DOI:** 10.1186/s42649-026-00144-8

**Published:** 2026-10-02

**Authors:** Soo-Yoon Hwang, Yeon-Seo Nam, Jaejin Hwang, Jonathon Schad, Jaekwang Lee, Chang-Beom Eom, Si-Young Choi

**Affiliations:** 1 Department of Materials Science and Engineering, POSTECH (Pohang University of Science and Technology), Pohang, 37673 South Korea; 2 Department of Physics, Pusan National University, Busan, 46241 South Korea; 3 Department of Materials Science and Engineering, University of Wisconsin-Madison, Madison, WI 53706 USA; 4 Center for Van der Waals Quantum Solids, Institute for Basic Science (IBS), Pohang, 37673 South Korea; 5 Department of Semiconductor Engineering, POSTECH, Pohang, 37673 South Korea

**Keywords:** Anatase TiO_2_, Surface engineering, Photoreactivity, Tin doping, Heterojunction

## Abstract

Anatase titanium dioxide (TiO_2_) is widely recognized for its superior photocatalytic capabilities due to its indirect band gap, low cost, and high chemical stability compared to other oxide semiconductors. Despite its advantages, TiO_2_ still suffers from intrinsic limitations, particularly rapid charge carrier recombination. To overcome these limitations, extensive research has focused on tailoring the surface structure of TiO_2_, particularly through morphology engineering that optimizes facet exposure and improves reactivity. In terms of photocatalysis, two surfaces can be mainly considered in anatase TiO_2_: (001) surface with notably high reactivity and (101) surface with lower reactivity; and thus the higher photocatalytic activity requires a higher proportion of the (001) surface, which contradicts to fact that the (001) and (101) surfaces have the surface energies of 0.90 J/m^2^ and 0.44 J/m^2^, respectively. We attempt Sn-doping to reduce the surface energy disparity and then stabilize the (001) surface. Our results demonstrate that Sn incorporation during growth stabilizes the (001) facet and promotes the formation of a (001)/(101) facet heterojunction. CL measurements further show suppressed radiative recombination relative to an undoped TiO_2_ thin film. We provide insights into the approach for achieving facet-selective surface stabilization through Sn incorporation.

**Supplementary Information:**

The online version contains supplementary material available at https://doi.org/10.1186/s42649-026-00144-8.

## Introduction

A photocatalyst is a type of catalyst that activates a chemical reaction upon exposure to light. The reaction involves irradiation with the energy exceeding band gap energy of materials, resulting in the excitation of electrons from the valence band to the conduction band, while simultaneously creating holes in the valence band. (Somiya 2013) The excited electrons, upon transferring energy externally, recombine with the holes. During the recombination phase, either oxygen or water, adsorbed on the materials’ surface, engages in a photocatalytic reaction with the excited electrons or holes. (Pant et al. 2019) Such photocatalytic properties have found extensive applications across various industries, including the decomposition of air and water pollutants, without the need for additional energy consumption.

Titanium dioxide (TiO_2_) stands out as a notable photoreactive material. Characterized by its non-toxicity, cost-effectiveness, and thermodynamic stability, TiO_2_ is not classified under the Globally Harmonized System (GHS). (Racovita 2022) Thus, it has been employed in various applications, traditionally serving as a white pigment in paints, ceramic glazes, and cosmetics. However, the discovery in 1972 that TiO_2_ could undergo photoelectrochemical water splitting under ultraviolet (UV) light conditions marked a pivotal moment, leading to an intensive exploration on photocatalytic properties of TiO_2_. (Fujishima, et al. 1972) TiO_2_ exhibits superior photocatalytic activity compared to other oxide semiconductors such as zinc oxide (ZnO), zirconium dioxide (ZrO_2_), and tin dioxide (SnO_2_), considering band gap, cost, and stability. (Ijaz, et al. 2021; Mills, et al. 1997; Hernández et al. 2015; Chou et al. 2007; Liao et al. 2012) Predominantly utilized in photocatalysis under UV light, TiO_2_ represents approximately 32% of the research in oxide photocatalysis. (Ijaz, et al. 2021) TiO_2_ exists in three polymorphic forms: anatase, rutile, and brookite. (Bencina et al. 2020) Among these, anatase TiO_2_ has an indirect band gap energy of 3.2 eV, which is slightly larger than the others (rutile: direct band gap of 3.0 eV, brookite: direct band gap of 3.13 eV). (Zhang et al. 2014; Scanlon, et al. 2013; Armakovic et al. 2023) In the case of rutile and brookite, only photon is emitted during the recombination of photoexcited electrons and holes in the direct band gap. In contrast, in anatase, the recombination of photoexcited electrons and holes is assisted by phonons due to the indirect band gap. The larger band gap and an indirect gap prolong a recombination time: the excited electrons in the indirect band gap of anatase TiO_2_ cannot directly recombine with holes, leading to an extended photoexcited electron–hole lifetime and photocatalytic reactions compared to rutile and brookite. (Zhang et al. 2014) Consequently, anatase TiO_2_ is acknowledged for its exceptional photocatalytic properties, especially in response to UV light. (Luttrell et al. 2014; Tang et al. 1995)

Beyond the band structure, a myriad of factors such as polymorphism, porosity, and surface energy significantly influence the photocatalytic properties of materials. (Luttrell et al. 2014; Cheng et al. 2019) In TiO_2_, surface morphology engineering has been predominantly carried out due to the photocatalytic reaction occurring on surfaces. (Dozzi et al. 2021; Yang et al. 2008; Lee et al. 2016; Han et al. 2009; Wang et al. 2016) For anatase-type TiO_2_ nanocrystals, photocatalytic reactivity is contingent upon the structural configuration of the surface, leading to variations in reactivity based on the exposed crystal plane. Specifically, in anatase TiO_2_, the (001) plane exhibits notably high reactivity, while the (101) plane demonstrates lower reactivity. (Han et al. 2009; Vittadini et al. 1998; Gordon et al. 2012; Gong, et al. 2005) The high reactivity of the (001) plane is attributed to its unique surface atomic structure, characterized by a low coordination number of exposed atoms and a dense concentration of Ti^4+^ atoms; On the (001) plane, the Ti coordination number is 5, while on the (101) plane, the Ti coordination number can be either 5 or 6. (Wang et al. 2016) Additionally, theoretical studies suggest that the O 2p state electrons on the (001) surface are more readily excitable, as deduced from electronic structure calculations. (Gong, et al. 2005; Yu et al. 2014; Sun et al. 2019; Lin et al. 2020) However, extensive empirical and theoretical researches have revealed that the (001) face of TiO_2_ possesses relatively high surface energy (surface energy, γ = 0.90 J/m^2^), rendering it unstable, whereas the (101) orientation is thermodynamically stable due to its lower surface energy (γ = 0.44 J/m^2^). (Vittadini et al. 1998; Gordon et al. 2012; Gong, et al. 2005; Chen et al. 2015; Lazzeri et al. 2001) The (101) orientation accounts for approximately 94% of the surface area, as indicated by the Wulff construction and theoretical calculations. (Lazzeri et al. 2001; Lai et al. 2019) This leads to a preference for the bipyramid morphology in TiO_2_ crystals. (Lazzeri et al. 2001)

However, it should be noted that the enhancement of photocatalytic activity is not linearly related to the increase in the exposure of the (001) surface on TiO_2_. In fact, overexposure of the (001) plane has been observed to lead to a gradual decline in photocatalytic activity due to easier recombination, as the electron-hole pairs are not spatially separated. (Lee et al. 2016; Wang et al. 2016) Studies have shown that a balanced configuration of (001) and (101) crystal facets is more preferable to solely maximizing the (001) facet. This is because exclusive dominance of the (001) facet may result in spatially conjoined electrons and holes, leading to their rapid recombination, thereby reducing the effectiveness of photocatalytic reactions. In photocatalytic reactions involving TiO_2_, photogenerated electrons migrate towards and accumulate on the lower energy (101) facet, while photogenerated holes congregate on the higher energy (001) facet, leading to their spatial separation. (S. W. Liu et al. 2011a, b; Zheng et al. 2011; Xiong et al. 2017) As such, achieving an optimal configuration of the facets is crucial for separating photogenerated electrons and holes effectively. Research indicates that the most favorable surface ratio for maximizing photocatalytic efficiency is achieved when the ratio of the (001) surface area (*S*_(001)_/*S*) is approximately 0.73. (Wang et al. 2016)

The synthesis of anatase TiO_2_ single crystals with a 47% exposure of the (001) facets was first reported by Yang et al. in 2008, employing fluorine. (Yang et al. 2008) The primary technique involves the adsorption of fluorine on the crystal surface using titanium tetrafluoride (TiF_4_) precursors, stabilizing the (001) facet relative to the (101) facet. Subsequent to these developments, the utilization of hydrofluoric acid (HF) as a capping agent in solvothermal synthesis has been established as an effective strategy. (Yang et al. 2008, 2009; Han et al. 2009; Liu et al. 2009a, 2010a, b, c, 2011a, b; G. Liu et al. 2010a, b, c a; Liu et al. 2009b) This method facilitates producing single-crystal TiO_2_ with as much as 89% exposure of the (001) facets. Consequently, adopting HF has become a widely adopted technique in the synthesis of TiO_2_ nanocrystals. However, the use of HF raises concerns due to its high toxicity and environmental impact, prompting a preference for HF-free synthesis processes. Despite this, there has been limited success in achieving fine control over facet orientation in HF-free methods. (Gordon et al. 2012; Yu et al. 2014; Lazzeri et al. 2001; G. Liu et al. 2010a, b, c b; Dai et al. 2009; Yu et al. 2012) Moreover, surface engineering of anatase TiO_2_ thin films still lack of studies, primarily due to the complexities arising from the robust interactions between the thin film and substrate. (Hwang et al. 2015; Shi, et al. 2017; Vasu et al. 2016; Kim et al. 2021; Islam et al. 2022; Lotnyk et al. 2007)

## Results and discussion

**Fig. 1:**
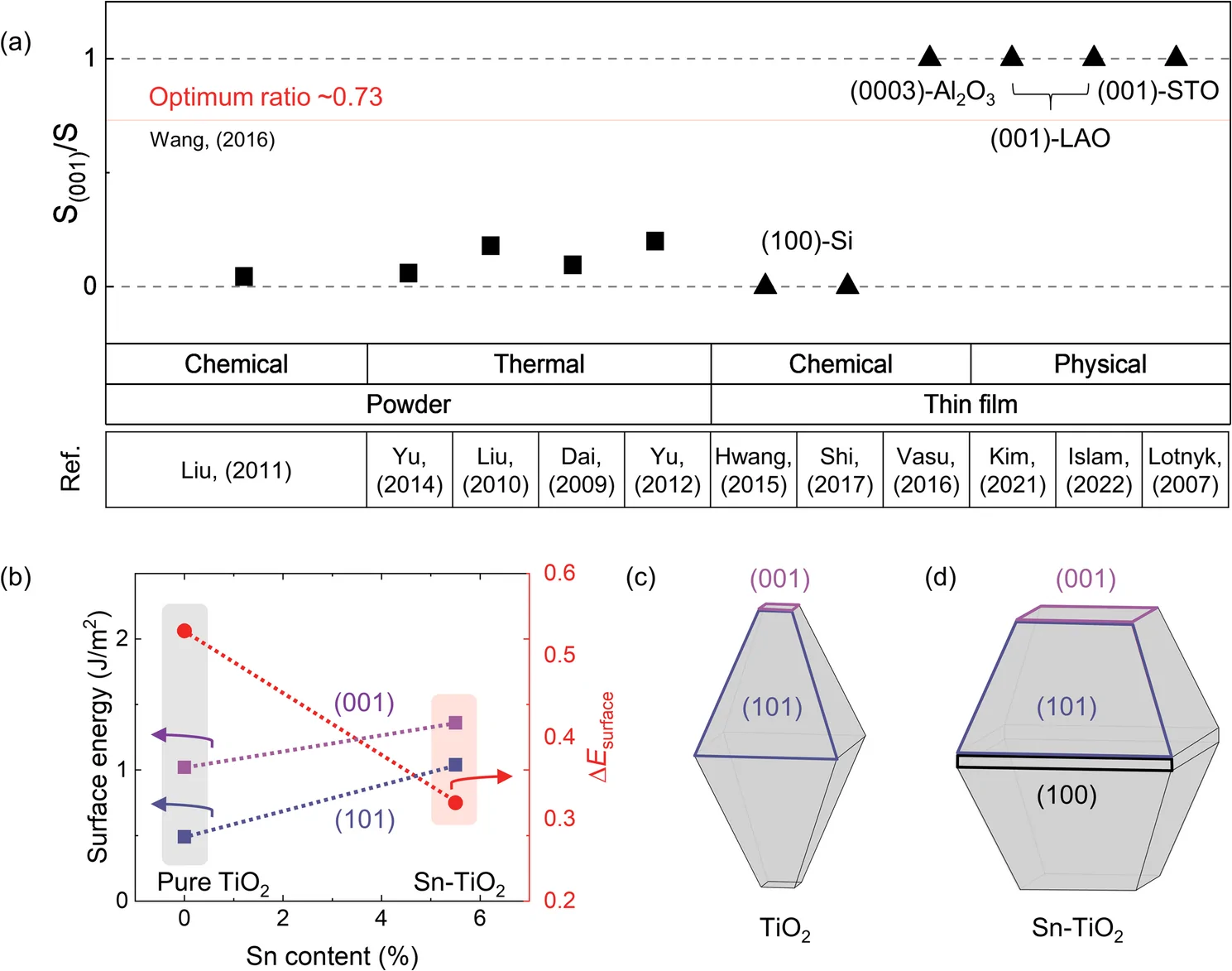
**a** Previously reported ratio of (001) plane (S_(001)_/S), where S is the total surface area in anatase TiO_2_. Optimum ratio of the (001) and (101) planes for the most efficient photocatalysts is marked by a red line. Each data point is labeled using the first author and publication year, and the corresponding references are provided in the reference list. **b** Surface energy (γ) of (001) and (101) planes in anatase TiO_2_ according to Sn doping. ΔE_surface_ is difference between the surface energies of (001) and (101). The schematics showing stable surface ratios of (**c**) pure TiO_2_ and (**d**) Sn doped TiO_2_. **b**-**d **are obtained by DFT calculation

Several methodologies have been proposed to adjust the exposure of the (001) plane in anatase TiO_2_, employing a range of chemical, thermal, and physical approaches in both nanopowder and thin film format. As illustrated in Fig. 1a, TiO_2_ nanopowders generally exhibit a preference for exposing the (101) plane, which is the most stable configuration, whereas thin films typically exhibit termination with either the (101) or (001) plane exclusively. (Gordon et al. 2012; Yu et al. 2014; Lazzeri et al. 2001; G. Liu et al. 2010a, b, c b; Dai et al. 2009; Yu et al. 2012; Hwang et al. 2015; Shi, et al. 2017; Vasu et al. 2016; Kim et al. 2021; Islam et al. 2022; Lotnyk et al. 2007) However, it shows that achieving the optimum ratio of 0.73, (Wang et al. 2016) marked by the red line in Fig. 1a, remains challenging. Hence, to achieve the optimal ratio and engineer the surface energy for maximizing the enhancement of photocatalytic properties in TiO_2_, we explored a new engineering method; Sn doping. To explore the role of Sn in the surface control of TiO_2_, we performed DFT calculations. The simulations ascertain the surface energies of both undoped and Sn doped TiO_2_ (Sn-TiO_2_) for the (101) and (001) planes, as depicted in Fig. 1b and Figure S2. For undoped TiO_2_, the (001) plane showed higher surface energy compared to the (101) plane, aligning with prior studies (Vittadini et al. 1998; Gordon et al. 2012; Gong, et al. 2005; Chen et al. 2015; Lazzeri et al. 2001), and exhibited a surface energy difference (Δ*E*_surface_) of 0.53 J/m^2^ between the two planes. However, the introduction of Sn dopants into TiO_2_ enhanced the stability of the (001) plane, reducing Δ*E*_surface_ to 0.32 J/m^2^. This decrease in Δ*E*_surface_ suggests that Sn doping mitigates the preferential exposure of specific planes, promoting the formation of diverse planes in anatase TiO_2_. Based on these computational results, we constructed predicted morphologies for both pure TiO_2_ and Sn-TiO_2_ (Fig. 1c and d). Consequently, Sn-TiO_2_ with a greater proportion of (001) planes is anticipated to demonstrate surface characteristics favorable for photocatalytic performance.

**Fig. 2:**
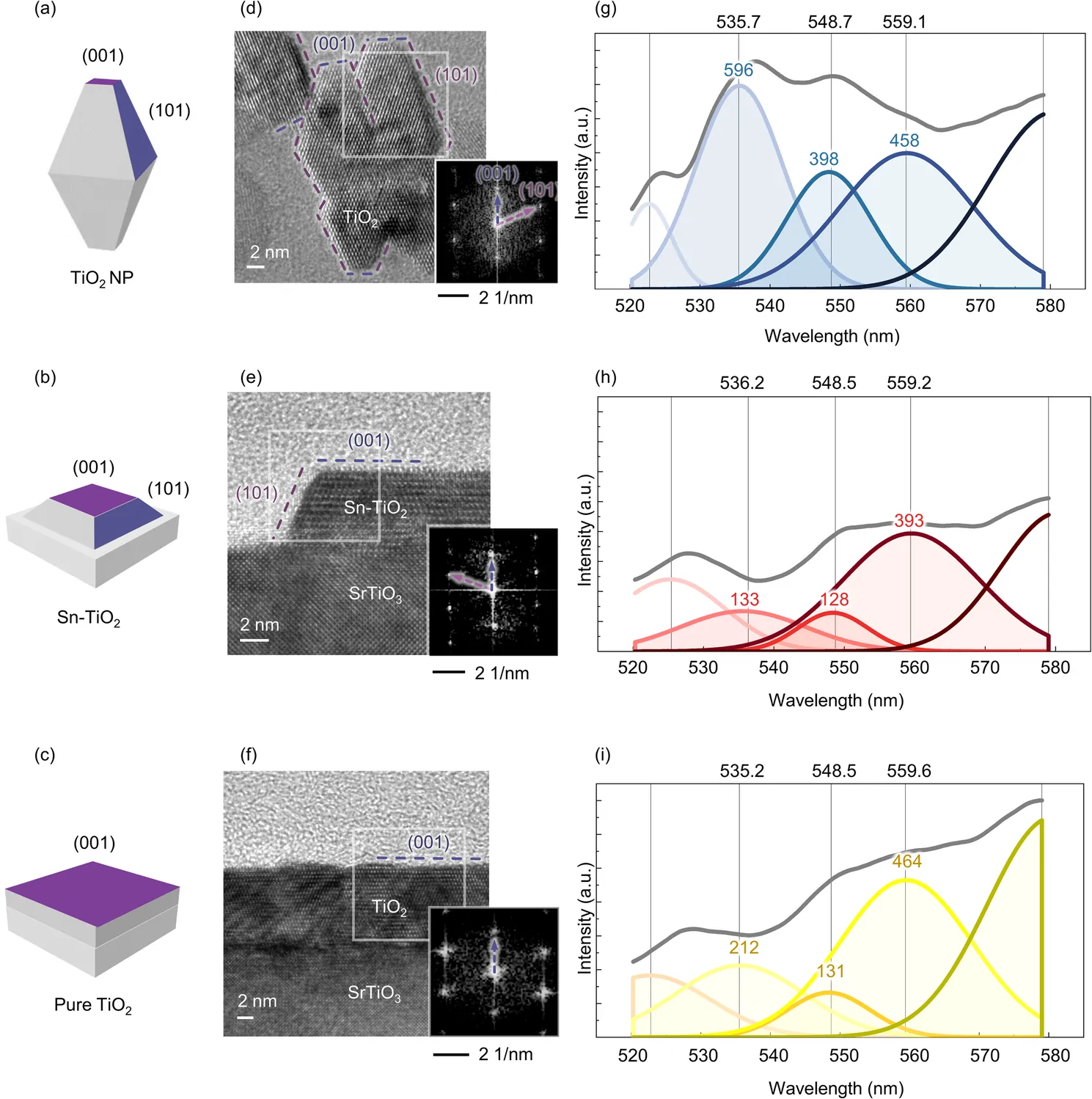
Morphological schematics of (**a**) TiO_2_ nanopowder, (**b**) Sn-TiO_2_ thin film and (**c**) TiO_2_ thin film. HR-TEM images of (**d**) TiO_2_ nanopowder, (**e**) Sn-TiO_2_ and (**f**) TiO_2_ thin film. Exposed surfaces of (**d**-**f**) are classified as (001) plane (blue dashed lines) and (101) planes (purple dashed lines), respectively. The TiO_2_ nanopowders predominantly have (101) planes, while the Sn-TiO_2_ thin film has surface termination of (101) and (001) planes in an optimal ratio (~ 0.73). Each inset of (**d**-**f**) is the FFT pattern of the HR-TEM images in the white boxes showing the surface orientations of TiO_2_ nanopowder, Sn-TiO_2_ and TiO_2_ thin film, respectively. CL measurement with the wavelength range from 520 nm to 580 nm are shown for (**g**) TiO_2_ nanopowder, (**h**) Sn-TiO_2_ and (**i**) TiO_2_ thin film, respectively. The gray lines indicate the obtained spectra, featuring deconvoluted bands centered at approximately 530 nm, 550 nm, 560 nm, and 580 nm

Based on the computational findings, we employed a novel synthesis approach to fabricate Sn-TiO_2_ thin films under HF-free conditions. This method combines chemical vapor deposition (CVD) and pulsed laser deposition (PLD), using titanium isopropoxide (TTIP). To investigate the influence of Sn doping on the morphological characteristics and surface preferences of anatase TiO_2_, we conducted a comparative analysis of three TiO_2_ variants: undoped TiO_2_ nanopowders, Sn-TiO_2_ thin films, and pure TiO_2_ thin films, as illustrated in Fig. 2a-c. The nanopowder particle size and the thicknesses of both thin films were adjusted to approximately 8 nm. HR-TEM images, as presented in Fig. 2d-f, display the respective morphologies of the TiO_2_ forms. The undoped TiO_2_ nanopowders exhibit truncated bipyramidal shapes (Fig. 2d and Figure S3), while the Sn-TiO_2_ film presents a flattened isosceles trapezoid profile in cross-section (Fig. 2e and Figure S4), with square-shaped islands around 100 nm in size (Figure S5a). The pure TiO_2_ thin film exhibits a highly crystalline, (001)-oriented anatase TiO_2_ structure, as confirmed by cross-sectional HR-TEM and the corresponding FFT (Fig. 2f). To discern the differences in surface exposure among these morphologies, we performed analyses of FFT patterns, derived from magnified HR-TEM images. The FFT images indicate the surface orientations of each sample, marked with blue and purple arrows. The FFT analysis revealed that the undoped TiO_2_ nanopowder, with the bipyramidal structure featuring a flattened top, predominantly expose the (101) planes, approximately 4.4 times more than the (001) planes, yielding a low *S*_(001)_/*S* ratio. In contrast, the Sn-TiO_2_ thin film attains the optimal *S*_(001)_/*S* ratio of approximately 0.73 between the (101) and (001) planes (Fig. 2e and Figure S4), whereas the pure TiO_2_ thin film predominantly exhibits the (001) orientation (Fig. 2f). The exposed surface ratio was quantified by combining the approximately square in-plane island morphology observed by AFM with the cross-sectional TEM geometry. Each island was approximated as a truncated square pyramid. The geometrical parameters a and b were measured, and the facet angle was θ ≈ 70°. Using these parameters, the surface ratio was calculated as *S(001)/S* = b²/{4[(a²/tan(θ)) + (a $\:\times\:$ b)] + b²}. Analysis of six Sn-TiO_2_ islands yielded *S(001)/S* values ranging from 0.68 to 0.76, with an average of 0.73 $\:\pm\:$ 0.03. This variation in surface ratio among the TiO_2_ nanopowder, Sn-TiO_2_ and TiO_2_ thin film indicates the potential for distinct photocatalytic responses in each form.

To assess the photocatalytic activity of pure TiO_2_ nanopowder and Sn-TiO_2_ thin film in relation to their respective surface ratios, CL measurements were performed. The CL measurement is sensitive and powerful technique to characterize defect states of anatase TiO_2_, including notable CL spectra originated from the radiative recombination of self-trapped excitons (STEs), as reported in previous studies. (Elrahoumi et al. 2023; Pallotti et al. 2017; Tachikawa et al. 2012; Nemashkalo et al. 2016) The band structure revealed by these spectra provides crucial insights into the photocatalytic properties of TiO_2_, highlighting the significant relationship between recombination time and photocatalytic effect. (Dadgostar et al. 2022) This is because rapid recombination (indicated by high CL spectrum intensity) of electron-hole pairs can hinder the separation of photogenerated charge carriers. Therefore, with a longer recombination time, charge carriers can persist for a longer duration (indicated by low CL spectrum intensity), thereby facilitating more chemical reactions and enhancing the photocatalytic effect.

We focused on the spectral range from 520 to 580 nm, characterized by five distinct peaks at specific wavelengths approximately 520 nm, 530 nm, 550 nm, 560 nm, and 580 nm. (Nair et al. 2014; Kovalevskiy et al. 2020; Lim et al. 2014; Amoresi et al. 2018) The characteristics of anatase TiO_2_ – such as longer Ti-Ti interionic distances, lower TiO_6_ octahedral coordination, and poor symmetry in anatase TiO_2_ – contribute to favor the green STE emission on defect states, (Pallotti et al. 2017; Mizoguchi et al. 2004) at about 2.3–2.5 eV. (Tachikawa et al. 2012; Nemashkalo et al. 2016) Consequently, in the Sn-TiO_2_ thin film, with its optimally tailored surface ratio, we anticipate a lower intensity in the CL signal at green wavelength region, indicating a delayed recombination process. Figures 2g-i illustrate the characteristic CL spectra of the TiO_2_ nanopowder, Sn-TiO_2_ thin film, and pure TiO_2_ thin film, respectively. Compared to pure TiO_2_ nanopowder, the signal intensity of Sn-TiO_2_ decreased by 77%, 67%, and 14%, respectively corresponding to 535 nm (2.3 eV) and 550 nm (2.25 eV), and 560 nm (2.2 eV). This reduction demonstrates the effective electron-hole separation facilitated by the (101)-(001) surface heterojunction, indicating a delayed recombination process. Compared to the pure TiO_2_ thin film, the signal intensity of Sn-TiO_2_ decreased by 37%, 2%, 15%, corresponding to 535, 548, and 559 nm, respectively. These findings imply that the Sn doping into TiO_2_ results in surface engineering and suppresses radiative recombination in TiO_2_. Notably, the suppressed radiative recombination is not simply attributed to the expanded (001) plane, but rather to the TiO_2_ thin film with the optimal surface ratio achieved through surface engineering. While CL measurements reveal the macroscopic photocatalytic effects of Sn-TiO_2_, understanding the atomic-scale mechanisms underlying these observations requires a more detailed structural analysis.

**Fig. 3:**
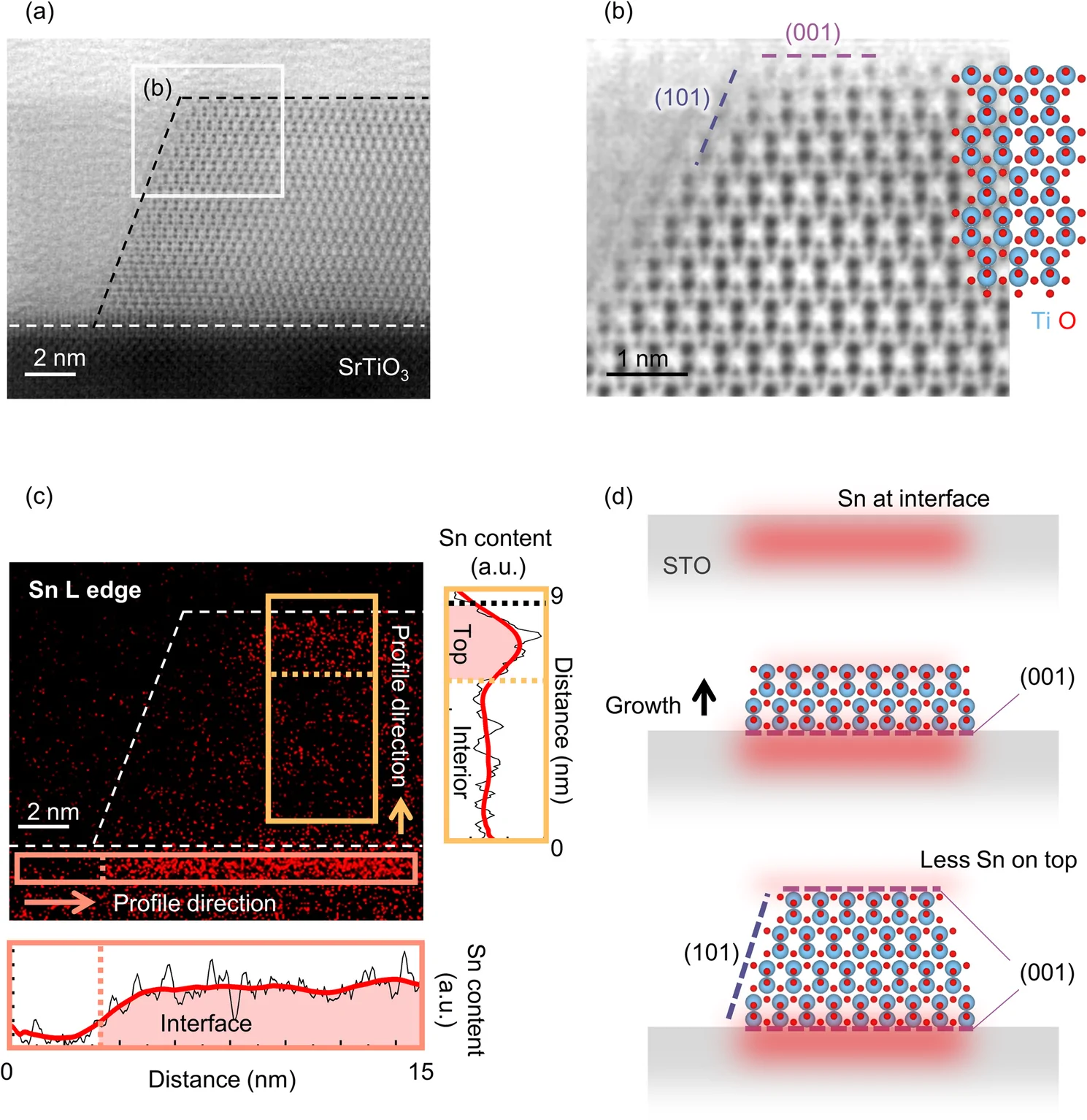
The atomic scale ABF-STEM images showing (**a**) the Sn-TiO_2_ thin film on SrTiO_3_ substrate and (**b**) the magnified edge of the Sn-TiO_2_. **c** The EDS map of Sn L edge in the same region of (**a**), and the chemical distribution profiles near interface (down) and along the film growth direction (right). **d** Schematics showing the role of Sn facilitating (001) planes during growth of Sn-TiO_2_ island

In order to explore the structural origin of the suppressed radiative recombination in Sn-TiO_2_ and Sn doping in detail, we employed STEM for atomic scale analysis. The ABF-STEM image presented in Fig. 3a reveals that the Sn-TiO_2_ exhibits a well-ordered atomic structure with minimal defects and distinct crystal orientations along both the (001) and (101) planes. Notably, as shown in Fig. 3b, the (001) and (101) planes are exposed without surface reconstruction, while the surface reconstruction for the (001) plane is frequently reported in anatase TiO_2_ lowering the surface energy due to an oxygen-deficient TiO_2-x_ termination, reducing Ti^4+^ to Ti^3+^. (Ma et al. 2022; Sotoudeh et al. 2014; Yuan et al. 2017) However, despite its energetic favorability, surface reconstruction on these planes in anatase TiO_2_ is often associated with a reduction in catalytic reactivity. Therefore, the clear surface without the reconstruction in Sn-TiO_2_ may contribute to the suppressed radiative recombination observed in CL measurements (Figs. 2g-i).

To further establish the structural impact of Sn doping, EDS was used for chemical composition mapping, as illustrated in Fig. 3c. A remarkable observation is the non-uniform distribution of Sn dopants within the film, as evidenced by the Sn distribution along the film growth direction (Fig. 3c (right) and near the interface region (Fig. 3c (down). Specifically, Sn dopants are predominantly located on the top surface of films, with a lower concentration in the interior (Fig. 3c (right); approximately 0.88 at% and 0.16 at% of Sn are present on the top surface and in the interior, respectively (as shown in Figure S6d and e). Moreover, the substrate-thin film interface exhibits an accumulation of Sn dopants (~ 1.05 at% Sn, in Figure S6f), in contrast to the substrate surface areas where Sn-TiO_2_ thin film growth was absent (Fig. 3c (down). The higher Sn concentration on the top surface has been observed because metal ions tend to preferentially occupy surface sites, where they achieve a lower electronic energy state compared to bulk sites. (Oropeza et al. 2013) As mentioned above, the suppressed radiative recombination observed in the Sn doped TiO_2_ thin film could be partly attributed by the effect of Sn on the surface. Previous studies have shown that appreciable photocatalytic enhancement by Sn doping occurs at higher Sn concentrations than the ~ 0.88 at% observed at our top surface. (Rajeswari et al. 2021; Chua et al. 2013) Therefore, although a localized direct effect of surface Sn cannot be excluded, it is unlikely to be the dominant origin of the enhanced photocatalytic activity. Instead, our DFT and microscopy results more directly support a growth-mediated role of Sn in stabilizing the (001) facet during film growth. Our EDS results further support the role of Sn in shaping the unique morphology of Sn-TiO_2_, as depicted in Fig. 3d. In our synthesis technique, SnO is utilized, but its inherent susceptibility to high temperatures (> 300 °C) leads to substantial vaporization, forming a Sn-enriched atmosphere during the synthesis process (800 °C). (Chen et al. 2003) Besides the established Sn-rich atmosphere, the continuous supply of SnO via the PLD source leads to localized accumulation of Sn on the SrTiO_3_ substrate (Figure S1).

The accumulated Sn preferentially forms self-assembled Sn-TiO_2_ islands on the surface of SrTiO_3_ substrates through the following process. (Shao, et al. 2018; Beniwal et al. 2021) First, Sn atoms form a wetting layer, which is a mono-atomic layer that forms on a substrate when the growth of thin films started. As the thin film grows, Sn clusters form to release the strain induced by the significant lattice mismatch between Sn (tetragonal, a = b=5.88 Å, c = 3.2 Å) and SrTiO_3_ (cubic, a = b=c = 3.905 Å). This considerable lattice mismatch, along with the difference in space groups, promotes the formation of Sn clusters on the SrTiO_3_ substrate. Our EDS data shows the Sn concentration at the interface between the substrate and the thin film is substantially higher. Second, this localized accumulation of Sn promotes TiO_2_ growth on SrTiO_3_ surface by decreasing the lattice mismatch through Sn substitution into Ti. (Oropeza et al. 2013) Sn (Sn^4+^ ionic radius ~ 69 pm) leads to the expansion of the TiO_2_ lattice from approximately 3.784 Å to 3.792 Å due to its larger ionic radius compared to Ti (Ti^4+^ ionic radius ~ 61 pm) (Figure S7). (Trung et al. 2023) This expansion reduces the lattice mismatch with SrTiO_3_ from approximately 3.1% to 2.8%, facilitating TiO_2_ growth on Sn-rich SrTiO_3_. (Shackelford, et al. 2001) Additionally, larger islands with lateral dimensions exceeding approximately 200 nm × 200 nm exhibit cracking, which further releases the lattice mismatch (Figure S5b). (Kim, et al. 2018)

In summary, a substantial accumulation of Sn on the substrate seems to contribute to stabilizing the (001)-island growth of TiO_2_. Further validation of this is provided by EDS data showing that Sn was not detected in substrate regions where TiO_2_ thin film growth did not occur, indicating that the higher lattice mismatch of approximately 3.1% in these Sn-deficient areas hinders TiO_2_ thin film growth. As the Sn-doped TiO_2_ thin film grows, surface diffusion of Sn stabilizes the growth of the (001) plane, contributing to the unique trapezoidal shape of the film, characterized by the narrowing of the (001) plane (Fig. 3d). This is consistent with previous computational results (Fig. 1b and c), demonstrating that the presence of Sn actively promotes the growth of the (001) plane in anatase TiO_2_. Our findings demonstrate how Sn doping modulates the surface energy, stabilized the highly reactive (001) plane, and promotes a surface structure associated with suppressed radiative recombination. From this delicate analysis, we conclude that Sn doping plays a crucial role in the selective stabilization of highly reactive (001) planes without reconstruction, shaping the trapezoidal morphology of Sn-TiO_2_, and promoting suppressed radiative recombination, which is favorable for photocatalytic performance.

## Conclusions

In short, we employed Sn doping as a strategy to optimize the surface ratio of TiO_2_. Initially, DFT calculations indicated that Sn doping could diminish the surface energy disparity between certain crystal facets, promoting the exposure of the (001) planes, which are thermodynamically less stable but exhibit heightened photocatalytic activity. Leveraging the insights, we adopted a synthesis method combining CVD and PLD that incorporates Sn. This approach yielded a TiO_2_ thin film with a distinctive square island-like morphology and a flat trapezoidal cross-section. CL measurements of the Sn-TiO_2_ films show reduced recombination-related emission relative to the undoped TiO_2_ nanopowders and thin film. Atomic-scale imaging revealed well-defined (001) and (101) planes without surface reconstruction. Furthermore, EDS mapping showed that Sn distribution was predominantly concentrated at the interface and the top surface of the films. Together with the DFT results, these observations indicate that Sn primarily acts during film growth by modifying the surface energetics and selectively stabilizing the (001) facet, thereby promoting the formation of a (101)/(001) facet heterojunction associated with suppressed radiative recombination.

## Experimental section

### Materials and synthesis (Figure S1)

Sn-TiO_2_ thin films were synthesized on TiO_2_-terminated SrTiO_3_ substrates using metalorganic pulsed laser deposition (MOPLD) which consisted of a custom pulsed laser deposition system (PLD) fitted with a showerhead for delivery of a heated metalorganic precursor to the sample surface. Film properties were monitored during deposition using reflection high energy electron diffraction (RHEED) for crystalline quality and Auger spectroscopy for surface chemistry. For MOPLD of Sn-TiO_2_, a SnO spark plasma sintered ceramic PLD target was used in conjunction with titanium tetraisopropoxide (TTIP) for a metalorganic TiO_2_ source. SrTiO_3_ substrates were prepared with TiO_2_-terminated, single unit cell stepped surfaces by first annealing in O_2_ at 1000 °C for 6 h and then etching for 30 s in 6:1 buffered hydrofluoric acid. The substrate was mounted to a heater using a colloidal silver solution before loading into the MOPLD system. Once the heater was loaded into the chamber, it was then heated to 800 °C in a 0.2 mTorr O_2_ atmosphere. A constant flow of TTIP was maintained during the deposition of the Sn-TiO_2_ thin film. To dope the film with Sn, a KrF (248 nm) ultraviolet excimer laser was focused onto the SnO PLD target and pulsed 250 times at 10 Hz SnO followed by 30 s of only flowing TTIP. This was repeated 25 times before cooling the substrate to room temperature. Pure TiO_2_ nanopowders, with an average size of around 8 nm, were purchased through Skyspring Nanomaterials, Inc.

### (Scanning) transmission electron microscopy

The TEM samples for the thin film were prepared by a dual-beam Focused Ion Beam system (Helios Nanolab, Thermo Fisher Co., USA) through the [010] projection. Cross-sectional high resolution (HR) TEM images were obtained using a 200 kV field-emission TEM (JEM-2100 F, JEOL, Japan). Electron diffraction patterns were acquired separately for each facet using the Fast Fourier Transform (FFT).

Cross-sectional STEM and EDS analyses were performed using a 200 kV STEM (JEOL ARM200F, JEOL, Japan) equipped with a 5th order probe-corrector (ASCOR, CEOS GmbH, Germany) and the dual $\:100\:{mm}^{2}$ energy dispersive X-ray spectrometer detector (JED-2300T EDS, JEOL, Japan) at Materials Imaging & Analysis Center of POSTECH in Republic of Korea. The electron probe for STEM observation was set to be ~ 70 pm; and the collection semi-angle was ranged from 54 to 216 mrad for High Angle Annular Dark Field (HAADF) imaging and from 13.5 to 27 mrad for Annular Bright Field (ABF) imaging. The raw STEM data were filtered to reduce the background scanning noise by using Local 2D Difference Filter (Filters Pro, HREM Research Inc., Japan). The EDS mapping signals were obtained during several tens of minutes by the multiple frame summation, up to 4000 frames of 256$\:\times\:$256 pixel-resolution; and the acquisition time per a single pixel was set to be $\:10\:\mu\:\mathrm{s}\mathrm{e}\mathrm{c}$. The EDS map was processed by the band-pass Wiener filter to reduce the background noise.

## Supplementary Information

Supplementary Material 1: Schematic of a Sn-TiO2 hybrid PLD process on the SrTiO3 substrate using TTIP and SnO target (Figure S1); DFT-calculated atomic structures of anatase TiO2 (top) and Sn-doped TiO2 (bottom) surfaces (Figure S2); STEM and STEM-EDS analysis of TiO2 nanopowder (Figure S3); Surface-ratio analysis of Sn-TiO2 thin film islands. (Figure S4); AFM results showing surface morphology (Figure S5); STEM-EDS analysis of Sn-TiO2 thin film (Figure S6); Cross-sectional HAADF-STEM image of the interface between Sn-TiO2 thin film and (001)-SrTiO3 substrate (Figure S7) (PDF).

## Data Availability

The datasets generated during the current study are available from the corresponding author on reasonable request.
