# Supplementary material for: Selective facet stabilization of anatase TiO2 by Sn doping
Source: Appl Microsc. 2026 Oct 2;56:24. doi: 10.1186/s42649-026-00144-8 (PMC13633235; doi:10.1186/s42649-026-00144-8)
Supplement: Supplementary file 1 [file 42649_2026_144_MOESM1_ESM.docx]

**Supporting Information**

**Selective Facet Stabilization of Anatase TiO_2_ by Sn Doping**

Soo-Yoon Hwang, Yeon-Seo Nam, Jaejin Hwang, Jonathon Schad, Jaekwang Lee, Chang-Beom Eom and Si-Young Choi^*^

**Table of Contents**

**Experimental section**

**Supplementary Information 1–7**

**Figure S1:** Schematic of a Sn-TiO_2_ hybrid PLD process on the SrTiO_3_ substrate using TTIP and SnO target.

**Figure S2:** DFT-calculated atomic structures of anatase TiO_2_ (top) and Sn-doped TiO_2_ (bottom) surfaces.

**Figure S3:** STEM and STEM-EDS analysis of TiO_2_ nanopowder.

**Figure S4:** Surface-ratio analysis of Sn-TiO_2_ thin film islands.

**Figure S5:** AFM results showing surface morphology.

**Figure S6:** STEM-EDS analysis of Sn-TiO_2_ thin film.

**Figure S7:** Cross-sectional HAADF-STEM image of the interface between Sn-TiO_2_ thin film and (001)-SrTiO_3_ substrate.

**References**

**Experimental Section**

**Cathodoluminescence (CL) measurement**

CL spectra of Sn-TiO_2_ and TiO_2_ nanopowder were obtained with a field-emission gun in a scanning electron microscope equipped with a high-sensitive CL monochromator (Monora 500i, Dongwoo optron, South Korea) in Materials Imaging & Analysis Center of POSTECH in Republic of Korea. The electron beam of the SEM is focused onto the specimen surface within a region of 300 nm$\times$500 nm, employing 10 kV and 40 $\mu A$ of probe current. The emitted CL spectra capturing up to 100 frames every 4 seconds were analyzed through a CL monochromator (a resolution of 0.5 nm per pixel, 1200 gratings per mm blazed at 300 nm, 20 $\mu$m entrance slit) with Andor charge-coupled device (CCD). All measurements are taken at room temperature. To determine the CL signals from TiO_2_, we also measured CL spectra of STO substrate and background to extract the corresponding signals.

**Density functional theory (DFT) calculations** (Figure S2)

All calculations were conducted using the Vienna ab initio simulation package (VASP) (Kresse, et al. 1996), incorporating the projector-augmented wave (PAW) method developed by Blochl (Blochl 1994) and implemented by Kresse and Joubert. The Perdew-Burke-Ernzerhof (PBE) generalized gradient approximation (GGA) was employed as the exchange correlation functional (Perdew, et al. 1996). Calculations were performed with a plane-wave basis set and an energy cutoff of 500 eV. A Γ-centered 4 × 4 × 1 k-point mesh was applied for the supercell slabs. The slab model was separated by a 15 Å-thick vacuum gap. Electronic convergence was achieved with a tolerance of 10⁻⁶ eV/cell, and structural relaxations were carried out until the forces on all atoms were less than 0.01 eV/Å. The surface energy ($\gamma$) was calculated using the following formula (Deringer, et al. 2015; Wang, et al. 2014; Zou, et al. 2016):

$\gamma=\frac{1}{2A}\left[ E_{slab}^{surf}-E_{slab}^{refer}-\sum_{\alpha} \Delta n_{\alpha}\mu_{\alpha} \right]$	(1)

Here, $A$ represents the surface area of the slab supercell, $E_{slab}^{surf}$ is the DFT total energy of the slab structure, and $E_{slab}^{refer}$ is the DFT total energy of the reference slab derived from the atomic energies of bulk TiO_2_. $\Delta n_{\alpha}$ denotes the difference in the number of atomic species $\alpha$ (Ti and Sn) compared to the reference slab. The DFT calculation was performed by using high-performance computing clusters in the Quantum Matter Core-Facility (QMCF) of Pusan National University.

Supporting Information 1

**Figure S1.** Schematic of a Sn-TiO_2_ hybrid PLD process on the SrTiO_3_ substrate using TTIP and SnO target.

Supporting Information 2

**Figure S2.** DFT-calculated atomic structures of anatase TiO_2_ (top) and Sn-doped TiO_2_ (bottom) surfaces: (a) (001) surface, (b) (101) surface, and (c) (100) surface.

Supporting Information 3

**Figure S3.** STEM and STEM-EDS analysis of TiO_2_ nanopowder. TEM images of (a) and (b) TiO_2_ nanopowder with different magnifications. (c) The HAADF-STEM image of TiO_2_ nanopowder and the atomic scale HAADF-STEM image of the surface terminated by (001) and (101) planes. STEM-EDS analysis (d-e) of TiO_2_ nanopowder; Ti K edge and Sn L edge, respectively. EDS spectra (f) taken along the marked the yellow boxes in the HAADF image in (d).

Supporting Information 4

**Figure S4**. Surface-ratio analysis of Sn-TiO_2_ thin film islands. (a) Schematic illustration of the geometrical parameters used for the calculation. (b) Cross-sectional TEM images showing the measured a and b values and the corresponding S(001)/S ratios.

Supporting Information 5

**Figure S5.** AFM results showing surface morphology. (a) Square shaped Sn-TiO_2_ islands viewed from the top. (b) Intensity profiles of selected islands indicated in (a).

Supporting Information 6

**Figure S6.** STEM-EDS analysis of Sn-TiO_2_ thin film. (a) Cross-sectional HAADF-STEM image and elemental EDS mapping of (b) Sn L edge and (c) Ti K edge. EDS spectra taken along the (d) surface, (e) film and (f) interface marked the yellow boxes in the HAADF image in (a).

Supporting Information 7

**Figure S7.** Cross-sectional HAADF-STEM image of the interface between Sn-TiO_2_ thin film and (001)-SrTiO_3_ substrate.

**References**

G. Kresse and J. Furthmuller, Phys Rev B 54, 11169 (1996).

P. E. Blochl, Phys Rev B 50, 17953 (1994).

J. P. Perdew, K. Burke, and M. Ernzerhof, Phys Rev Lett 77, 3865 (1996).

V. L. Deringer and R. Dronskowski, Angew Chem Int Edit 54, 15334 (2015).

J. F. Wang, J. W. Liu, Y. Xu, J. Wu, B. L. Gu, and W. H. Duan, Phys Rev B 89, 125308 (2014).

Y. C. Zou, Z. G. Chen, F. T. Kong, E. Z. Zhang, J. Drennan, K. Cho, F. X. Xiu, and J. Zou, Nanoscale 8, 19383 (2016).
